# Renoprotective Effects of Goreisan via Modulation of RAAS Activity, Oxidative Stress, and AQP2 Trafficking in a Rat Model of Nephrotic Syndrome

**DOI:** 10.3390/biomedicines14010008

**Published:** 2025-12-19

**Authors:** Mao Shimizu, Shunsuke Goto, Satoshi Yamatani, Kazuo Sakamoto, Keiji Kono, Hideki Fujii

**Affiliations:** Division of Nephrology, Kobe University Graduate School of Medicine, Kobe 650-0017, Japan; nebosukeomachan@yahoo.co.jp (M.S.); sgoto@med.kobe-u.ac.jp (S.G.); rdozuran@yahoo.co.jp (S.Y.); cazoo0202@gmail.com (K.S.); kkono@med.kobe-u.ac.jp (K.K.)

**Keywords:** AQP2, Goreisan, nephrotic syndrome, oxidative stress, renin–angiotensin–aldosterone system

## Abstract

**Background/Objectives**: We evaluated Goreisan, a traditional Chinese medicine, for its effects on nephrotic syndrome in a rat model. **Methods:** Male Sprague–Dawley rats underwent right nephrectomy at 5 weeks of age, followed by adriamycin administration (5 mg/kg) at 6 and 8 weeks of age to induce nephrotic syndrome. At 10 weeks, rats were divided into three groups: vehicle (control), Goreisan 0.5 g/kg (GL), and Goreisan 1.0 g/kg (GH). Goreisan was administered daily for 4 weeks. At 14 weeks, blood, urine, mRNA expressions, and kidney histopathology were analyzed. Data were analyzed using one-way ANOVA followed by Tukey–Kramer post hoc testing. **Results:** Goreisan prevented worsening kidney function, with reduced glomerular and tubulointerstitial damage, lower systemic and intrarenal 8-hydroxy-2′-deoxyguanosine levels, and lower plasma aldosterone levels and expression of intrarenal renin–angiotensin–aldosterone system (RAAS)-related factors. Urine volume significantly increased in GL and GH groups compared with the control group. In the GH group, urine volume increased markedly (Δ urine volume: 10.0 ± 2.6 mL/day), whereas it tended to decrease in the Vehicle group (Δ urine volume: −1.3 ± 2.5 mL/day). Urine osmolality was lower in the GH group, with a larger decrease in Δ urine osmolality (−616.3 ± 132.8 mOsm/L). These changes occurred without an increase in urinary sodium excretion, suggesting an aquaretic effect independent of natriuresis. Creatinine clearance (CCr/kg) declined markedly in the Vehicle group but was significantly preserved in the GH group (Δ CCr/kg: −2.2 ± 0.19 vs. −0.7 ± 0.28), indicating renoprotective effects. No differences were found in serum arginine–vasopressin levels. Real-time PCR and immunohistochemical staining showed no significant differences in aquaporin (AQP) mRNA expression (AQP1, AQP2, AQP3, and AQP4), but AQP2 localization to the apical membrane in the collecting ducts was reduced with Goreisan treatment. **Conclusions:** Goreisan demonstrates kidney-protective and diuretic effects in nephrotic syndrome, potentially through reducing systemic oxidative stress, modulating RAAS activation, and altering AQP2 trafficking.

## 1. Introduction

Chronic kidney disease (CKD) is a global health issue, with an estimated 850 million patients as of 2019 [1]. Patients with CKD have a high incidence of cardiovascular diseases (CVD), which are associated with poor prognosis and reduced quality of life [2,3]. Among CVD, heart failure is known to be the most common in CKD [4]. This may be due to the impaired regulation of body fluids, one of the crucial functions of the kidneys, leading to sodium and water retention as kidney function deteriorates. Consequently, loop diuretics, such as furosemide, are often used during fluid retention; however, these diuretics can reduce the intravascular volume, potentially decreasing renal blood flow and worsening kidney function [5]. Additionally, hyperuricemia is a commonly observed side effect. Thus, diuretics that have less influence on renal function are considered desirable in CKD.

Goreisan is a traditional herbal medicine, composed of five ingredients, that has been used for centuries in China, Japan, and Korea. It has traditionally been used to treat disorders of fluid homeostasis, such as edema and diarrhea. In clinical practice in Japan, this medication is available in the form of extract granules and is officially indicated for conditions such as nephrotic syndrome, uremia, edema, and diabetes mellitus, and it is also commonly prescribed for patients with CKD. More recently, Goreisan has been reported to exert renoprotective effects in several experimental studies [6,7,8]. In an adriamycin-induced kidney injury model, Goreisan effectively reduced the amount of urinary protein and ameliorated the diminished urine output [8]. Furthermore, tubulointerstitial injury in a hypertensive model and in a folic acid-induced CKD model was alleviated by Goreisan [6,7]. In the kidney, water reabsorption in the collecting duct is regulated by aquaporin-2 (AQP2). Apical membrane trafficking of AQP2 is controlled by vasopressin-dependent phosphorylation at multiple serine residues in its C-terminal region. Phosphorylation of Ser256 enables apical targeting of AQP2, whereas dephosphorylation at Ser261 and phosphorylation at Ser264 and Ser269 regulate its apical localization and stabilization [9]. Dysregulation of AQP2 trafficking has been implicated in impaired urinary concentration and fluid imbalance in various kidney diseases, including nephrotic syndrome. Although Goreisan is expected to reduce proteinuria, promote diuresis, and mitigate tubulointerstitial damage in the field of nephrology, detailed data and mechanisms have not been elucidated. In particular, the precise mechanisms by which Goreisan induces aquaretic diuresis—especially its effects on aquaporin-2 (AQP2) trafficking and renin–angiotensin–aldosterone system (RAAS) modulation in nephrotic syndrome—remain insufficiently understood.

We aimed to clarify the diuretic effects and impact of Goreisan on kidney lesions. To resolve this issue, we created a nephrotic syndrome rat model, exhibiting abnormal fluid balance and proteinuria, and investigated the diuretic and renoprotective effects of Goreisan.

## 2. Materials and Methods

### 2.1. Animals and Experimental Protocol

Male SD rats were sourced from CLEA Japan Inc. located in Tokyo, Japan. These rats were kept in environments where light and temperature were regulated, and they had unlimited access to food and water. When they reached 5 weeks old, the rats underwent a right nephrectomy while under anesthesia, which was induced by the intraperitoneal injection of medetomidine, midazolam, and butorphanol. Subsequently, adriamycin (ADR) (Aspen Japan, Tokyo, Japan) (5 mg/kg body weight) was administered via the tail vein at 6 and 8 weeks of age. From 10 weeks of age, Goreisan (Tsumura & Co, Tokyo, Japan) was administered orally for 4 weeks at a low (0.5 g/kg body weight; GL group: *n* = 6) or a high dose (1.0 g/kg body weight; GH group: *n* = 6). The control group received water orally. Each animal was assigned an identification number and allocated to the three groups using a simple randomization method based on these numbers. Goreisan used in this study was a commercially available Kampo extract powder composed of five herbal ingredients: *Alisma tuber* (tuber of Alisma orientale Juzepczuk), *Atractylodes lancea rhizome* (rhizome of Atractylodes lancea De Candolle), *Polyporus sclerotium* (sclerotium of Polyporus umbellatus Fries), *Poria sclerotium* (sclerotium of Poria cocos Wolf), and *Cinnamon bark* (Cinnamomum cassia Blume) [10]. The extract powder was suspended in distilled water and administered orally via gastric gavage. At 14 weeks old, the rats were euthanized while under anesthesia with pentobarbital sodium (Tokyo Chemical Industry Co., Ltd., Tokyo, Japan). Euthanasia was performed by intraperitoneal injection of an overdose of pentobarbital sodium. After euthanasia, a thoracotomy and complete exsanguination were carried out to confirm death. Excessive weight loss, impaired ambulation, or labored breathing was defined as a humane endpoint. In line with the ARRIVE guidelines, all possible measures were taken to alleviate animal discomfort during the experiments [11,12].

This investigation was conducted in alignment with the Guide for the Care and Use of Laboratory Animals, as provided by the National Institutes of Health. The study protocol received approval from the Institutional Animal Ethics Committee at the Kobe University Graduate School of Medicine (Permit No.: P170301).

### 2.2. Blood and Urine Measurements 

Blood and urine samples were collected and stored at −80 °C following centrifugation at 3000 rpm. Serum creatinine (Cr), albumin (Alb), and urinary sodium levels were measured using the Fuji Dri-Chem 3500 system (Fujifilm Japan, Tokyo, Japan). We analyzed urinary Cr, protein, and plasma arginine vasopressin (AVP) levels at SRL Corporation (Tokyo, Japan). Serum and urinary osmotic pressures were measured at FUJIFILM Wako Pure Chemicals Corporation (Osaka, Japan). An enzyme-linked immunosorbent assay (ELISA) kit (Japan Institute for Control of Aging, Shizuoka, Japan; RRID: AB_2895008) was used to detect urinary excretion of 8-hydroxy-2′-deoxyguanosine (8-OHdG), a sensitive marker of oxidative stress. The detection limit was 0.5 ng/mL, and the intra-assay CV was less than 10%. Serum aldosterone levels were quantified using an ELISA kit (ab136933, Abcam, Cambridge, UK; RRID: AB_2895004). The detection limit was 4.7 pg/mL, and the intra-assay CV was approximately 4.4–6.6%.

### 2.3. Blood Pressure Measurements

Blood pressure was recorded using a tail-cuff plethysmograph (Model MK-2000; Muromachi Kikai Co., Ltd., Tokyo, Japan). Measurements were conducted after an adequate acclimatization period to minimize the potential stress-related artifacts. Systolic blood pressure was determined by taking multiple readings for each rat at baseline and at the end of the study period.

### 2.4. Immunohistochemical and Histomorphological Analyses

For histopathological analysis, kidney tissue samples were preserved in 10% formaldehyde. These samples underwent dehydration using 70% ethanol at ambient temperature and were then embedded in paraffin blocks. The blocks were later deparaffinized and prepared for immunohistochemical staining. The sectioning and staining tasks were carried out by Morphotechnology Co., Ltd., located in Sapporo, Japan.

The extent of stiffening in the glomerular tufts, which serves as an indicator of disease progression, was evaluated on periodic acid-Schiff (PAS)-stained paraffin sections using a semiquantitative scoring method. For each animal, the glomerular score was calculated as the average of 100 glomeruli and examined under light microscopy (Olympus Corporation, Tokyo, Japan) at a magnification of 400×. The severity of glomerulosclerosis was graded on a scale from 0 to 3, as follows [13]: grade 0, no change; grade 1, mild segmental hyalinosis, sclerosis, or vacuolization affecting 25% of the glomerular tuft; grade 2, moderate segmental hyalinosis, sclerosis, or vacuolization involving 25–50% of the tuft; grade 3, diffuse hyalinosis, sclerosis, or vacuolization involving more than 50% of the tuft. The index for each animal was represented as the mean of all scores. Tubulointerstitial damage was also assessed using a semiquantitative scoring system on PAS-stained paraffin sections at 100× magnification. Ten fields per kidney were randomly selected to determine the tubulointerstitial damage score, with changes graded on a scale from 0 to 3 as follows: grade 0, no change; grade 1, lesions affecting less than 25% of the area; grade 2, lesions affecting 25–50% of the area; and grade 3, lesions involving more than 50% of the area.

8-OHdG formation was evaluated using anti-8-OHdG monoclonal antibodies raised in rats (Japan Institute for Control of Aging, Shizuoka, Japan). AQP2 expression was stained using anti-AQP2 monoclonal antibodies. To assess AQP2 localization and trafficking, 10 fields of view were examined on AQP2-stained paraffin sections at 10× magnification. We counted the amount of collecting duct cells within each observation area and the percentage of cells positive for AQP2 in the apical membrane of the collecting duct. To assess oxidative stress, the number of 8-OHdG-positive cells in the glomeruli and tubules was counted in 20 and 10 random microscopic fields, respectively. All evaluations were conducted in a blinded manner to ensure objectivity.

### 2.5. RNA Extraction and Real-Time PCR

RNA was isolated from rat kidney tissues using an ISOGEN kit (Wako Pure Chemicals Industries Ltd., Osaka, Japan) in accordance with the manufacturer’s guidelines. Complementary DNA (cDNA) was created using the qPCR RT Kit (TOYOBO Co., Ltd., Osaka, Japan) and an oligo-dT primer, following the instructions provided by the manufacturer. The synthesized cDNA was stored at −80 °C until it was ready for further examination through qPCR. The expression levels of messenger RNA (mRNA) were assessed using the Applied Biosystems 7500 Real-Time PCR System (Thermo Fisher Scientific, Waltham, MA, USA) with the SYBR Green Assay and Thunderbird SYBR qPCR Mix (TOYOBO Co., Ltd.), adhering to the manufacturer’s protocol. The analysis employed the relative quantification method available in the Applied Biosystems 7500 Real-Time PCR Software v2.0.6. Following this, the relative mRNA expression levels in the samples were adjusted to the expression of glyceraldehyde 3-phosphate dehydrogenase (GAPDH) mRNA. The primers used for PCR are listed in Table 1.

### 2.6. Bioimpedance

We assessed fluid volume using a bioelectrical impedance technique with the ImpediVET device (ImpediMED Ltd., Brisbane, Australia). Four 25-gauge needles (Nipro Corporation, Osaka, Japan) were inserted along the dorsal midline of the animal at the anterior margin of the orbit, anterior margin of the ear, sacral/caudal junction, and base of the tail. The needle tips were bent at a 90° angle for proper positioning. Measurements were performed both at baseline and endpoint, and the frequency range of the ImpediVET device was 4–1000 kHz. We obtained five consecutive measurements, and the data were analyzed using the manufacturer’s bioimpedance software (Version 1.0.2.7) to calculate TBW, ECF, and ICF.

### 2.7. Statistical Analysis

The data are presented as the mean ± SEM. Normality was assessed using the Shapiro–Wilk test. Since only the glomerular scores did not follow a normal distribution, the Kruskal–Wallis test was used for these ordinal histological data, followed by post hoc pairwise comparisons with Bonferroni correction. To compare groups, a one-way analysis of variance was utilized, followed by the Tukey–Kramer test. Depending on the situation, either Pearson’s correlation coefficient or Spearman’s rank correlation coefficient was employed to examine the relationships between variables. *p* < 0.05 was considered statistically significant. All statistical analyses were performed using IBM SPSS Statistics version 23.0 (Chicago, IL, USA).

## 3. Results

### 3.1. Animal Characteristics and Biochemical Measurements

**Figure 1:**
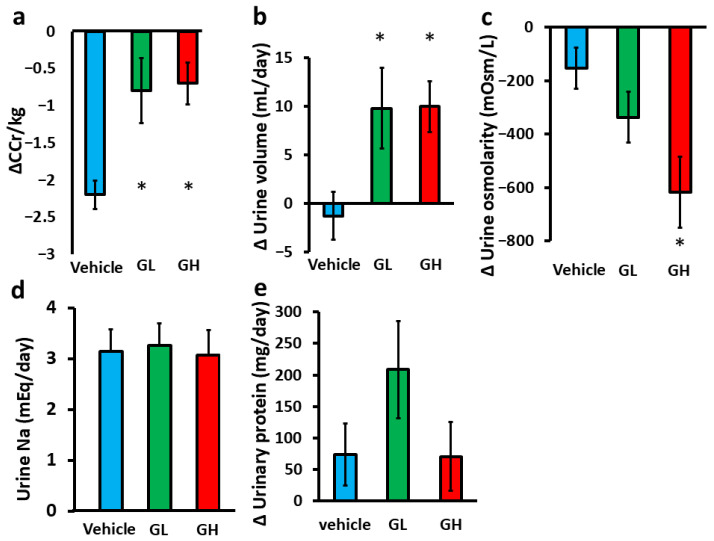
Effects of Goreisan administration on renal function and urinary biochemistry. Light blue indicates the Vehicle group (*n* = 6), green indicates the GL group (*n* = 6), and red indicates the GH group (*n* = 6). (**a**) Change in kidney function after Goreisan administration. (**b**) Change in urine volume after Goreisan administration. (**c**) Change in urine osmolality after Goreisan administration. (**d**) Urinary sodium excretion after Goreisan administration. (**e**) Change in urinary protein levels after Goreisan administration. *; versus vehicle; *p* < 0.05. CCr, creatinine clearance; GL, Goreisan low dose; GH, Goreisan high dose; Na, sodium.

Table 2 shows the baseline animal characteristics and biochemical data at 10 weeks of age for each group. All groups had proteinuria, with no significant differences between them. Additionally, systolic blood pressure, renal function, urine volume, osmolality, or urinary sodium excretion showed no intergroup differences. Figure 1 shows the effects of Goreisan administration on renal function and urinary biochemistry. After 4 weeks of Goreisan treatment, the decline in creatinine clearance (CCr) was significantly attenuated (Figure 1a), and urine volume was significantly increased (Figure 1b) in the GL and GH groups compared with the control group. In the biochemical evaluation of urine, urine osmolality was significantly decreased in the GH group, with a similar decreasing trend noted in the GL group (Figure 1c). However, sodium excretion and urinary protein excretion were not significantly different among the groups (Figure 1d,e).

### 3.2. Histological Evaluation

**Figure 2:**
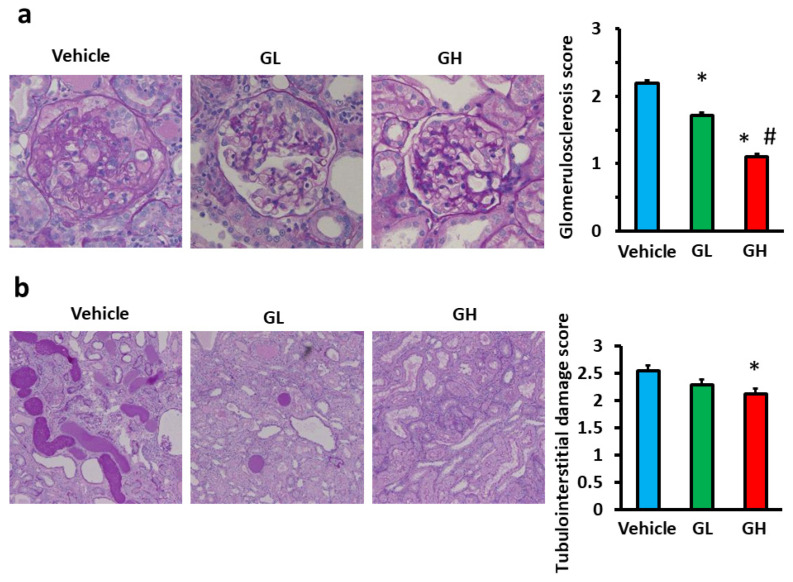
Evaluation of renal tissue damage. Light blue indicates the Vehicle group (*n* = 6), green indicates the GL group (*n* = 6), and red indicates the GH group (*n* = 6). (**a**) Representative images of glomeruli with PAS staining (magnification, ×400) and glomerulosclerosis score. (**b**) Representative images of the tubules with PAS staining (magnification, ×100) and tubulointerstitial damage score. *; versus vehicle; *p* < 0.05, #; versus GL; *p* < 0.05. GL, Goreisan low dose; GH, Goreisan high dose.

We evaluated the effects of Goreisan on renal tissue histologically. Figure 2 presents the microscopic images of the glomeruli and tubules for each group. The glomerulosclerosis score in the GL and GH groups was significantly lower than in the control group, with the GH group displaying a significantly lower score than the GL group (Figure 2a). Similarly, the tubulointerstitial damage score in the GH group was significantly reduced relative to the control group (Figure 2b).

### 3.3. Evaluation of Oxidative Stress

**Figure 3:**
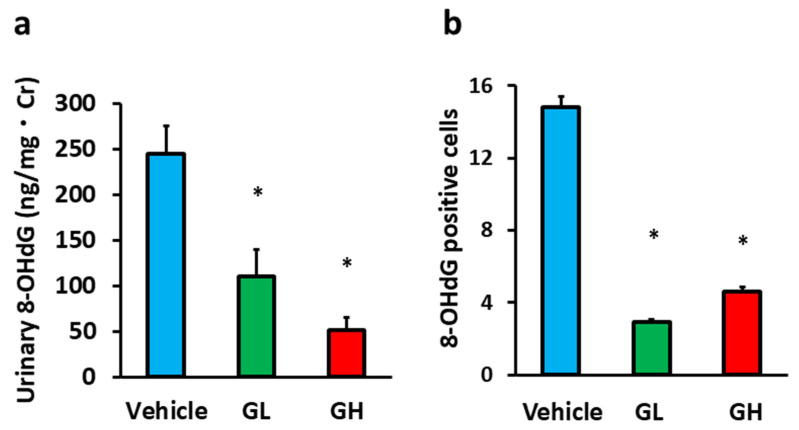
Evaluation of the oxidative stress. Light blue indicates the Vehicle group (*n* = 6), green indicates the GL group (*n* = 6), and red indicates the GH group (*n* = 6). (**a**) Urinary 8-OHdG levels. (**b**) Number of 8-OHdG-positive cells in the glomeruli. *; versus vehicle; *p* < 0.05. GL, Goreisan low dose; GH, Goreisan high dose; 8-OHdG, 8-hydroxy-2′-deoxyguanosine.

Urinary 8-OHdG level measurement and 8-OHdG-positive cell analysis in the renal tissue were used to assess oxidative stress. Urinary 8-OHdG levels in the GL and GH groups were significantly lower than in the control group (Figure 3a). Similarly, the number of 8-OHdG-positive cells in the glomeruli in the Goreisan-treated groups was significantly reduced (Figure 3b).

### 3.4. Effects on the Renin–Angiotensin–Aldosterone System (RAAS)

**Figure 4:**
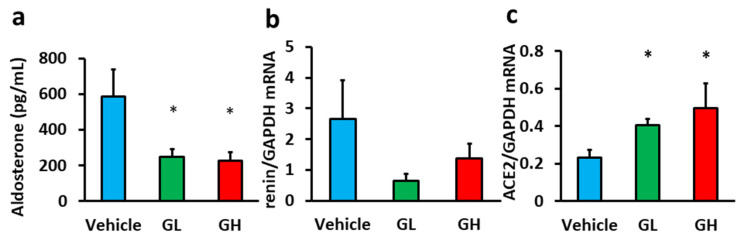
Evaluation of the renin–angiotensin–aldosterone system. Light blue indicates the Vehicle group (*n* = 6), green indicates the GL group (*n* = 6), and red indicates the GH group (*n* = 6). (**a**) Serum aldosterone concentration. (**b**) mRNA expression of renin in the renal tissue. (**c**) mRNA expression of ACE2 in the renal tissue. *; versus vehicle; *p* < 0.05. GL, Goreisan low dose; GH, Goreisan high dose; ACE2, angiotensin-converting enzyme 2.

We evaluated the effects of Goreisan on the RAAS. Serum aldosterone levels in the GL and GH groups were significantly lower than in the control group (Figure 4a). Renin mRNA expression in the renal tissue showed a decreasing trend in the Goreisan groups (Figure 4b), where ACE2 mRNA expression was significantly increased (Figure 4c).

### 3.5. Effects on the Body Fluid Composition

**Figure 5:**
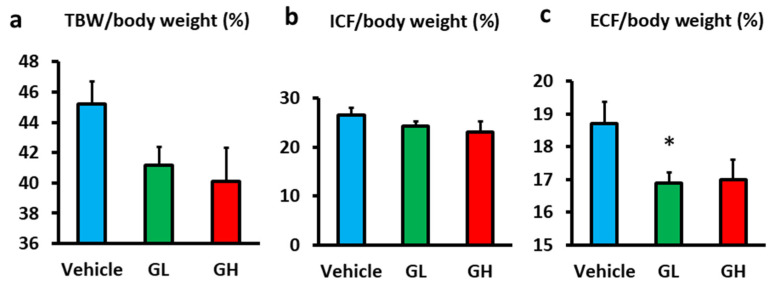
Evaluation of body fluid composition using bioelectrical impedance. Light blue indicates the Vehicle group (*n* = 6), green indicates the GL group (*n* = 6), and red indicates the GH group (*n* = 6). (**a**) TBW/body weight (%). (**b**) ICF/body weight (%). (**c**) ECF/body weight (%). *; versus vehicle, *p* < 0.05. GL, Goreisan low dose; GH, Goreisan high dose; TBW, Total body water; ICF, Intracellular fluid; ECF, Extracellular fluid.

Bioelectrical impedance was used to evaluate body fluid composition. Although the differences in total body water (TBW)/body weight were not statistically significant, the Goreisan groups tended to have lower TBW/body weight than the control group (Figure 5a). Further fluid compartment analysis revealed no significant differences in the intracellular fluid (ICF) among the groups (Figure 5b). However, extracellular fluid (ECF/body weight) tended to be lower in the Goreisan group, with a significant reduction observed in the GL group (Figure 5c).

### 3.6. Expression of Aquaporin (AQP) in the Renal Tissue

**Figure 6:**
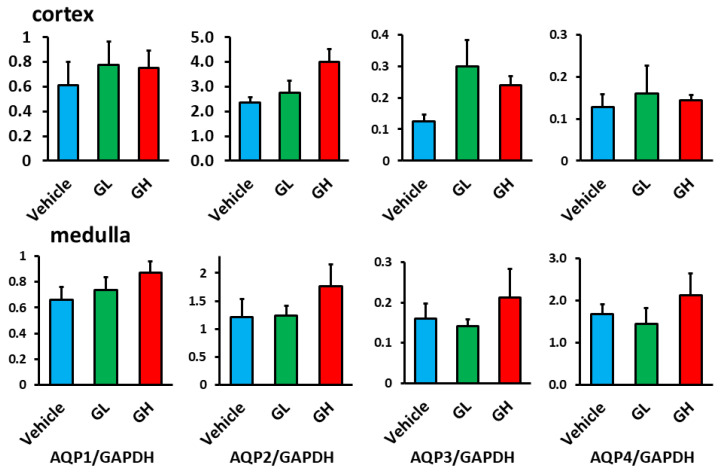
mRNA expression of AQP1–4 in the renal cortex and medulla. Light blue indicates the Vehicle group (*n* = 6), green indicates the GL group (*n* = 6), and red indicates the GH group (*n* = 6). GL, Goreisan low dose; GH, Goreisan high dose; AQP1, aquaporin-1; AQP2, aquaporin-2; AQP3, aquaporin-3; AQP4, aquaporin-4.

**Figure 7:**
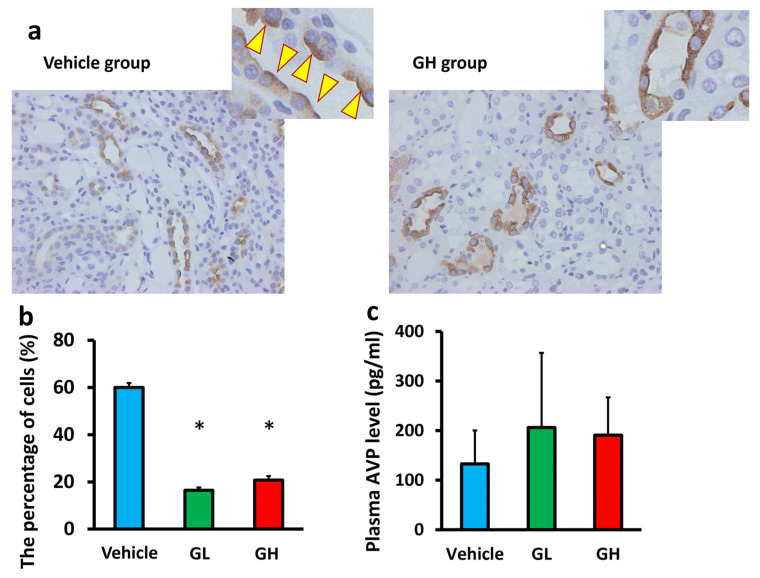
Localization of AQP2 in the collecting tubules and plasma AVP levels. Light blue indicates the Vehicle group (*n* = 6), green indicates the GL group (*n* = 6), and red indicates the GH group (*n* = 6). (**a**) Localization of AQP2. AQP2 was predominantly localized on the luminal side of the collecting tubules (arrowhead). (**b**) Percentage of cells with apical membrane localization of AQP2. (**c**) Plasma AVP levels GL, Goreisan low dose; GH, Goreisan high dose; AQP2, aquaporin-2; AVP, arginine vasopressin. *; versus vehicle; *p* < 0.05.

We examined AQP expression in the renal tissue. mRNA analysis showed that the expression of AQP1–4 in the renal cortex and medulla did not significantly differ between the Goreisan-treated groups and the control group (Figure 6). Although AQP1, which is constitutively expressed in the proximal tubules and descending thin limbs, and basolateral AQPs (AQP3 and AQP4) in collecting duct principal cells showed no significant changes, immunostaining revealed distinct alterations in the localization of AQP2, a vasopressin-regulated water channel expressed on the apical membrane of collecting duct principal cells. In the control group, AQP2 was predominantly localized on the luminal side of the tubules. In contrast, apical membrane localization of AQP2 was significantly reduced in the GL and GH groups compared with the control group (Figure 7a,b). Plasma AVP levels did not differ significantly among the three groups (Figure 7c).

## 4. Discussion

Our study showed that the decline in renal function was suppressed in the Goreisan-treated groups compared with the control group, with less severe glomerulosclerosis and tubulointerstitial damage in the renal tissue. Both systemic and intrarenal oxidative stress, and RAAS activation, were alleviated. Additionally, Goreisan administration significantly increased the urine volume; however, urine osmolality decreased, and no differences were observed in urinary sodium excretion. These findings suggest that the effect of Goreisan is primarily aquaretic rather than natriuretic, consistent with the observed reduction in apical localization of AQP2, indicating inhibition of AQP2 trafficking and promotion of water excretion.

Several in vitro and in vivo studies have demonstrated that Goreisan alleviates kidney injury [6,7,8,14,15]. In vitro, Goreisan mitigates mesangial cell proliferation induced by hyperglycemia and tubular epithelial cell damage caused by cisplatin [14,15]. In vivo studies using other CKD models, such as hypertensive rats and folic acid-induced CKD mice, reported that Goreisan reduces tubulointerstitial injury [6,7]. Another study in an adriamycin-induced kidney injury model [8], similar to our nephrotic model, showed that Goreisan improved urine output, decreased urinary protein excretion, and prevented podocyte injury. Consistently, in our study, Goreisan prevented the decline in CCr, indicating protection against glomerular and tubulointerstitial injuries. These findings histologically indicate that Goreisan may exert a renoprotective effect.

The RAAS plays a critical role in CKD progression [16], and numerous studies have demonstrated the renoprotective effects of RAAS inhibitors [17,18,19]. We hypothesized that RAAS inhibition is one of the potential mechanisms underlying the renoprotective effects of Goreisan. Indeed, previous studies showed that Goreisan reduces intrarenal angiotensin II levels increased by adriamycin [8] and suppresses systemic and intrarenal RAAS in a renal artery stenosis model rat [20]. In our study, Goreisan decreased plasma aldosterone levels and increased renal ACE2 mRNA expression. It should be noted that the observed decreases in RAAS-related markers in our study may reflect not only a direct inhibitory effect of Goreisan but also a secondary effect resulting from amelioration of renal injury, and both interpretations are possible.

Oxidative stress significantly contributes to kidney damage [21]. Goreisan administration significantly reduced urinary 8-OHdG excretion and decreased 8-OHdG expression in the kidney tissue. Similarly, in a folic acid-induced kidney injury model, Goreisan suppressed renal 4-hydroxynonenal (4-HNE) upregulation [7], suggesting antioxidative effects.

Goreisan has been reported to correct the water balance [22]. The mechanism of correcting water balance may involve the effect of Goreisan on AQP2 in the renal tubules [7,10,20,23]. A study using renal artery stenosis model rats and Sprague–Dawley (SD) rats has shown that Goreisan reduces AQP2 and vasopressin V2 receptors (V2R) expression in the kidneys [20]. In addition, Goreisan reduces AQP2 protein expression in renal tubular cells subjected to hypertonic solutions [23]. Conversely, Goreisan tended to increase AQP2 mRNA expression in the kidneys, which had been decreased due to kidney damage, in a folic acid-induced kidney injury model [7]. Our study showed that Goreisan did not alter AQP2 mRNA expression in the kidneys, indicating that its effect on AQP2 expression in the kidney may vary depending on the CKD condition. Thus, we hypothesized that changes in AQP2 localization might be involved and conducted further investigations. When renal tubular cells are exposed to hypertonic solutions, AQP2 becomes concentrated on the apical membrane, but Goreisan alleviates this localization [23]. Accumulating evidence indicates that AQP2 trafficking to the apical membrane is tightly regulated by post-translational modifications, particularly phosphorylation at specific serine residues in the C-terminal region, such as Ser256, Ser261, Ser264, and Ser269, which play distinct roles in apical targeting and membrane retention [9]. Among these regulatory mechanisms, in mouse kidneys, desmopressin increases AQP2 phosphorylation at serine 269, which was inhibited by Goreisan [10]. Consistent with these findings, our study showed that Goreisan reduced the apical membrane localization of AQP2, indicating that one of the mechanisms by which Goreisan induces aquaretic diuresis is by inhibiting the translocation of AQP2 to the apical membrane.

In clinical practice, loop diuretics are widely used for conditions such as heart failure and nephrotic syndrome, but their use is associated with adverse events including electrolyte disturbances, worsening renal function, and activation of the RAAS. In contrast, Goreisan exhibits aquaretic effects with a lower risk of electrolyte imbalance and demonstrated RAAS suppression in this study. Along with the histological renoprotective effects observed, Goreisan may represent a relatively safe therapeutic option for these patients.

**Figure 8:**
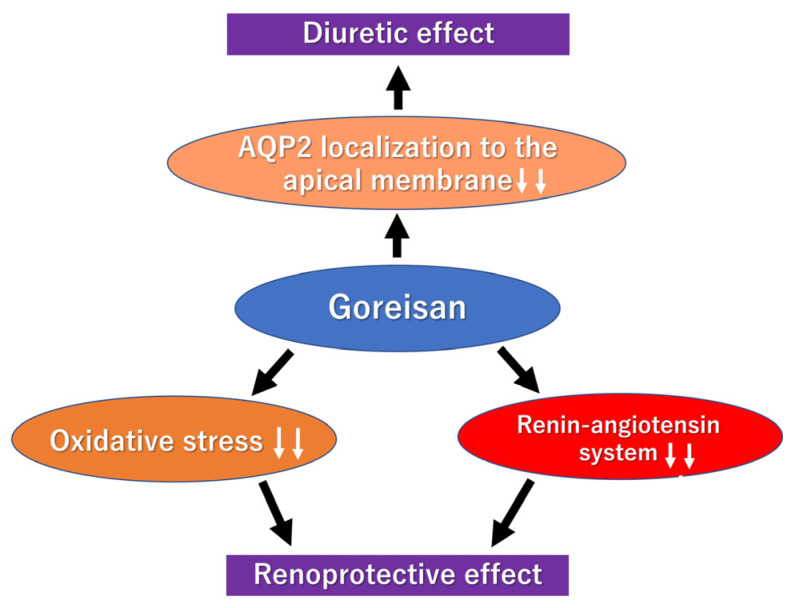
Schematic overview of Goreisan’s renoprotective and aquaretic effects.

The key findings of the present study are summarized in Figure 8, which illustrates the proposed mechanisms by which Goreisan exerts renoprotective effects, modulates AQP2 trafficking, and promotes aquaretic diuresis.

This schematic illustrates the key mechanisms of Goreisan observed in the present study. Goreisan inhibited apical membrane trafficking of AQP2 in the renal collecting ducts, promoting aquaretic diuresis. It also reduced intrarenal oxidative stress and suppressed the renin–angiotensin–aldosterone system (RAAS), contributing to renoprotection. All arrows in the figure are solid and indicate the direction of effect, either stimulation/increase or inhibition/decrease. These changes summarize the main findings of this study.

This study has several limitations, which also provide directions for future research. First, the sample size was relatively small (n = 6 per group), which may limit statistical power. Second, only male rats were used, so potential sex-related differences could not be evaluated. Additionally, the adriamycin nephropathy model does not fully replicate human nephrotic syndrome, and therefore clinical translation should be approached cautiously. Notably, we were able to demonstrate Goreisan-induced changes in AQP2 trafficking, highlighting its potential mechanism of action, although Ser269 phosphorylation of AQP2 and plasma renin levels were not assessed in this study. These aspects represent important areas for further investigation, which will help to deepen understanding of Goreisan’s renoprotective effects.

## 5. Conclusions

Goreisan may exert renoprotective effects by modulating the intrarenal RAS and oxidative stress-related pathways. Additionally, Goreisan induces aquaretic diuresis by reducing the apical membrane localization of AQP2 in the renal tubules. These findings suggest that Goreisan may be useful as an adjunctive therapy for conditions associated with kidney injury and fluid retention, although further studies are needed to clarify its clinical applicability.

## Figures and Tables

**Table 1:** Primer sequences used for PCR.

Gene	Forward Primer (5′→3′)	Reverse Primer (3′→5′)
AQP1	GCTGTCATGTATATCATCGCCCAG	AGGTCATTTCGGCCAAGTGAGT
AQP2	CAGTAGAAATCCGTGGGGACC	CGTCGGTGGAGGCAAAGAT
AQP3	GAGTTGATGAACCGTTGCGG	TTGATGGTGAGGAAGCCACC
AQP4	ACCTGTGATAGCACCTTGCC	GCAGGGAGGTCCACACTTAC
Renin	CTGTGCATACTGGCTCTCCA	GGCTTGGCCTAAAACTAGGG
ACE2	CCCAGAGAACAGTGGACCAAAA	GCTCCACCACACCAACGAT
GAPDH	TGGGAAGCTGGTCATCAAC	GCATCACCCCATTTGATGTT

**Table 2:** Animal characteristics at baseline.

	Vehicle (*n* = 6)	GL (*n* = 6)	GH (*n* = 6)
Body weight (g)	252.8 ± 29.2	271.8 ± 15.9	304.2 ± 18.3 *
Blood pressure (mmHg)	106.9 ± 18.5	108.5 ± 9.5	113.0 ± 5.1
Alb (g/dL)	2.0 ± 0.2	2.3 ± 0.4	2.4 ± 0.6 *
BUN (mg/dL)	45.4 ± 13.5	47.0 ± 20.2	35.8 ± 13.9
Creatinine (mg/dL)	0.54 ± 0.13	0.60 ± 0.17	0.47 ± 0.14
Na (mEq/L)	135.7 ± 2.1	138.0 ± 2.5	136.3 ± 1.6
Plasma osmolality (mOsm/L)	302 ± 9.8	293.0 ± 16.3	295.0 ± 12.8
Urine volume (mL/day)	29.1 ± 9.5	19.3 ± 3.7	16.5 ± 7.0
Urine protein (mg/day)	509.2 ± 142.7	413.8 ± 142.2	362.9 ± 121.5
Urine osmolarity (mOsm/L)	954.3 ± 333.3	1085.7 ± 185.7	1437.7 ± 524.0
CCr/kg	3.4 ± 0.3	3.2 ± 0.5	3.8 ± 0.3
Urine Na (mEq/day)	3.8 ± 0.5	3.9 ± 0.6	3.9 ± 1.1

Alb, albumin; BUN, blood urea nitrogen; Na, sodium; CCr, creatinine clearance; * *p* = 0.005 vs. vehicle.

## Data Availability

The data supporting the findings of this study are not publicly available but are available from the corresponding author upon reasonable request.

## Abbreviations

ACE2	Angiotensin-converting enzyme 2
ADR	adriamycin
Alb	albumin
AQP	aquaporin
AVP	arginine vasopressin
CCr	creatinine clearance
cDNA	complementary DNA
CKD	chronic kidney disease
Cr	creatinine
CVD	cardiovascular disease
ECF	extracellular fluid
ELISA	enzyme-linked immunosorbent assay
ERA	European Renal Association
GAPDH	glyceraldehyde 3-phosphate dehydrogenase
GH	high dose Goreisan
GL	low dose Goreisan
ICF	intracellular fluid
mRNA	messenger RNA
NOX4	NADPH oxidase 4
PAS	periodic acid-Schiff
RAAS	renin–angiotensin–aldosterone system
TBW	total body water
V2R	vasopressin V2 receptors
4-HNE	4-hydroxynonenal
8-OHdG	8-hydroxy-2′-deoxyguanosine
